# Ethical considerations of social media use for surgeons

**DOI:** 10.1007/s00464-026-12770-0

**Published:** 2026-04-13

**Authors:** Carmen Fong, Kathleen Park, Claire Rosen, Alberto Ferreres, Marian McDonald

**Affiliations:** 1 Hemorrhoid Centers of America, Atlanta, GA USA; 2 Robert Wood Johnson Barnabas University Hospital, New Brunswick, NJ USA; 3 Hospital of the University of Pennsylvania, Philadelphia, PA USA; 4 University of Buenos Aires, Buenos Aires, Argentina; 5 LG Health Physicians/Penn Medicine, Lancaster, PA USA

**Keywords:** Social media, Ethics, Patient-provider communication, Privacy, Physician disclosure

## Abstract

**Background:**

Social media has become an increasingly prevalent platform for exchanging health information, facilitating professional networking, promoting education, and fostering public engagement. For surgeons, its benefits—rapid dissemination of knowledge, community building, conference amplification, advocacy, and recruitment—coexist with heightened ethical risks, including breaches of confidentiality, blurred professional boundaries, misinformation, conflicts of interest, and inequities in access. This SAGES Ethics Committee white paper provides an ethics-focused overview of surgeon social media use and offers practical recommendations aligned with core bioethical principles, incorporating previous work from the SAGES Social Media Committee, the SAGES Facebook Taskforce, and the SAGES Ethics Committee.

**Methods/Approach:**

We synthesize existing professional guidance and the peer-reviewed literature on social media in healthcare and surgery to identify recurring ethical dilemmas across stakeholder groups (surgeons, patients, institutions, and society). We organize these issues using the four principles of biomedical ethics—autonomy, beneficence, non-maleficence, and justice—and translate them into actionable standards for professional conduct, content stewardship, and institutional oversight.

**Results:**

Key ethical domains include the following: (1) professionalism and identity management in blended personal/professional spaces; (2) confidentiality and the Health Insurance Portability and Accountability Act of 1996 (HIPAA)-informed safeguards when sharing clinical images, videos, and case discussions; (3) disclosure and conflict-of-interest management in self-promotion, marketing, endorsements, and “non–evidence-based” content; (4) boundaries in patient interaction, emphasizing that social media should not be used for direct patient-provider communication in lieu of secure, trackable clinical platforms; (5) respect for patient privacy, including a general expectation that clinicians should not search patients’ social media absent compelling, disclosure-supported exceptions (e.g., emergent identification needs); (6) consent standards for recording or posting media involving patients or clinicians; (7) equity considerations, recognizing that reliance on social platforms can worsen disparities for individuals lacking access or digital health literacy; and (8) societal-level implications such as clinical trial recruitment, crowdsourcing, misinformation correction, wellness harms from excessive use, and emerging risks/opportunities from artificial intelligence (AI)-enabled amplification and data mining.

**Conclusions:**

Ethical social media engagement by surgeons is feasible and often beneficial when guided by transparency, accuracy, confidentiality protection, boundary maintenance, and equity. We recommend clear disclosures, separation of personal/professional accounts when feasible, institutional and society-level monitoring frameworks for official messaging, strict consent and de-identification standards for clinical content, avoidance of social media as a clinical communication channel, and ongoing review of AI-driven changes to platform dynamics and privacy risk.

Social media has become a source of viable and valuable information for patients and clinicians that can no longer be ignored. Certain professional societies have guidelines for using social media, but a comprehensive overview is required to explore the medical and public health ethics of using social media. This white paper examines what constitutes professional and ethical behavior when it comes to surgeons using social media and surveys the ethical considerations around surgeons’ choices regarding social media. For the purpose of this paper, social media is separate and distinct from the general information that can be found on the Internet and is defined as the use of various online platforms through which users create communities to share information, ideas, personal messages, and other content [1].

Multiple papers have addressed the use of social media for the dissemination of information during the COVID-19 pandemic. People were in lockdown and unable to obtain health information in person from their healthcare providers. This ‘infodemic’ changed the optics of using social media for health information. Before 2019, it was unclear whether medical professionals should be using social media platforms at all. Now, with multiple platforms that have channels dedicated to health information (“MedTwitter,” “DokTok”), professionals must address the proper, ethical use of these channels. Avoiding social media interaction entirely is no longer an option. Some societies even encourage program directors to be better acquainted with social media to model appropriate behavior for their residents. However, misuse of social media can lead to severe consequences such as breaches of patient confidentiality. ‘Approved’ uses of social media include networking with colleagues and follow-up discussions during and after scientific conferences. Where the line becomes blurry is when providers: (a) use social media as a means of self-promotion, (b) use social media to educate the public, (c) use social media to communicate directly with patients, (d) the ethical implications of using patient videos in educational and public forums on social media, and (e) the regulation of content publicly and privately available on social media.

## Ethical principles addressed

Within this paper, the ethics of social media use within medicine will be addressed on multiple levels. Many of the considerations noted here, along with outlined recommendations, should align with the four major bioethical principles of autonomy, non-maleficence, beneficence, and justice [2]. Patient and provider consent, in some capacity, is essential to the sharing of information, and the privacy of both providers and patients must be protected. Any posted content must do no harm to patients or providers, and although this may not be the first goal, it cannot be missed. Content that is posted or shared should be beneficial and even educational for both patients and providers. Finally, all people should have equal access to the information shared on social media when used for patient education. Lack of a computer, smartphone, or digital health literacy should not act as a barrier to a patient’s ability to access vital health information or clinical trials. Providers should consider patients’ access when choosing to disseminate information online. The depth of ethical issues regarding digital technology and the use of social media for case-based video education is beyond the scope of this immediate paper but will be addressed in future SAGES publications.

## Physician use of social media

*Physicians’ use of social media for self-promotion or marketing is not inherently unethical, but it should be carefully considered and conflicts of interest should be disclosed.*

Overall, physician use of social media has been beneficial for networking and education from both a professional and patient standpoint; however, there have been concerns about confidentiality and the increasingly less distinct boundary between the personal and professional on blended social media profiles [3]. This increased social media use by physicians has also resulted in a noted increase in the number of citations for those authors who have a marketable social media presence (X and Facebook): an over 2.5-fold increase on Google and Scopus within three years after publication [4].

*Physicians should include a visible disclosure that their views do not necessarily represent the institution, organization, or society, unless otherwise stated by the institution, organization, or society.*

The rate of physician use of social media has been increasing over the last ten years, with one survey of over 4,000 physicians showing that more than 90% use social media personally, and about 65% use social media for professional purposes. One study of physicians from top US academic hospitals finds younger and female physicians are more likely to have a social media account. Surgeons and older physicians tend to have more followers [14].

While the trend has been to separate personal and professional accounts, the line has become increasingly blurred as social media platforms evolve. For example, platforms such as LinkedIn, Sermo, and Doximity are conceived for professional use only, with Sermo and Doximity being only accessible by registered physicians. However, physicians may post whatever they want without censorship, and topics unrelated to their professional work, such as politics, might be addressed. When these posts appear related to the physician’s place of work, there may be a conflict of interest. Studies have shown that institutional social media use and ‘branding’ increase the likelihood that patients will seek out that institution for their care [5]. This may be extrapolated to include physicians using social media for self-promotion, though no study exists yet to verify whether this is, in fact, effective. Social media may also be damaging to a physician’s professional image.

When used for self-promotion, the interests of the affiliated institution and the patient must be considered. In general, while a physician may claim, and rightfully acknowledge, appropriate association with a professional society, organization, or institution, the physician should not leverage such an association for their personal gain. Physicians using personal accounts should have disclaimers in their profiles stating that their views do not necessarily represent the society/organization/institution, and no harm to any individual patient may be caused.

In addition, surgeons promoting services or procedures that deviate from societal guidelines’ standards of care, or goods and products that may not be evidence-based, should do so with the disclaimers that their opinions are their own and may not be based on available evidence.

In thinking about the ethical principle of justice when considering physicians’ use of social media for self-promotion, it should be stressed that the diversity of physicians could be misrepresented. Physicians may be limited by access to social media resources, or limited by time to create content when not performing their clinical duties. In addition, those who are represented on social media may or may not have the clinical acumen as portrayed by their social media presence, a concept that patients seeking providers may not understand. In these situations, caveat emptor, or “buyer beware,” applies.

*Physicians’ use of social media for education and public dissemination should be subject to self-verification of accurate information that protects patient and provider confidentiality.*

Various media platforms now exist for educational content to enhance medical education, as well as forums for practitioners to share knowledge regarding practices, updates, and personal career development. Regarding social media accounts, multiple professional societies have pages/groups with moderators regulating, updating, and overseeing discussions. A 2019 SAGES Statement on Closed Social Media Groups (Facebook) endorsed the professional use of CSMG platforms for education and dissemination of clinical information with the proviso that ethical and compliant use of the platform, including informed consent, remains the responsibility of the posting physician. Legal implications remain unclear as to when the content and the commentary of contributors may place the physician in a compromised position. The question of new techniques posted, and replicating what is viewed on social media without specific training, also remains an important question. There is a risk of inaccurate content or lack of regulation of accurate content. Assigned oversight by content experts for these members-only closed groups may be an important aspect of using these discoverable platforms for education as well as discussion of complicated cases. Legal protection should be a priority of national surgical societies whose members rely increasingly on these types of posting for educational purposes—both for the patient (informed consent and complication management) and for the surgeon contributor. “Surgeons and patients embracing CSMG for quality improvement and optimized outcomes should be legally protected. SAGES foresees the use of this type of platform continuing to grow.”[15]

SAGES continues to have a significant presence in social media on X/Twitter and Facebook and other private and public forums. While content in these official social media postings is monitored, the content of the private groups or more public forums such as X or Facebook groups remains unexplored.

These platforms, in turn, can serve as a verified, reliable source of information to millions of people [6]. Initially instituted during the COVID-19 pandemic, many medical conferences turned toward virtual platforms with regular use of video conferencing, and even though many have returned to in-person meetings, the virtual component often persists [7, 8]. By removing the travel and opportunity costs associated with in-person meetings, attendance levels have overall increased. On the other hand, this benefit must be weighed against the in-person networking opportunities through multi-center collaborations, congregation opportunities for industry partners, and hands-on workshops. Outside of conferences, standardized webinars and specialty-specific podcasts can disseminate information with a broader and more diverse reach than previously possible [8]. On an even wider scale, public health organizations can not only spread initiatives but also rebut misinformation on social media in a timely and widespread manner [9].

*Social media used for educational purposes should be used with institutional oversight. Institutions and major surgical societies should dedicate staffing for monitoring and approval of tagged social media statements.*

Social media is increasingly used for the recruitment of residency applicants. One program noted that prospective applicants ranked Twitter and Instagram within the top three most commonly used resources to determine the culture of a residency program [10]. Use of these platforms is increasingly important for today’s resident applicants and the information on these platforms must be accurate representations of the program. Applicants will expect defined data points (number of cases, board pass rates, etc.) in addition to resident testimonials, after-residency placements, etc. This may also be subject to public scrutiny which may define an institution’s academic reputation. Care should be taken to ensure accuracy and protect the residents’ privacy.

The use of YouTube videos in surgical training has increased dramatically in recent years. In fact, YouTube videos have been cited as one of the most used platforms for medical students, surgical trainees, and practicing surgeons, for surgical education.

This content is not regulated, peer-reviewed, nor part of a private group such as on Facebook (which may allow for commentary and correction) and thus exposes the learners, and the public, to a wide variety of unregulated quality of content. Lima et al. performed a systematic review of 24 articles addressing social media as a tool in surgical education and found that the advantages of using social media for surgical education should be considered, although the quality of the material still needs to be vetted to ensure it is evidence- and guideline-based [11].

As per the American College of Surgeons’ Qualifications of the Responsible Surgeon, “a surgeon’s release of material to communications media or nonprofessional publications should be designated only for education and public information [and] such releases must be accurate” [12]. A commonly used educational tool in social media is the sharing of interesting cases, mystery diagnoses, or thought-provoking imaging. Discussion forums utilizing videos and images allow for a space to share knowledge and consult with other professionals [6]. Though these cases can certainly be of value, practitioners must consider institutional and societal guidelines and remove all identifying information. Of note, physicians report confidentiality to be the greatest barrier to professional social media use among non-users [13]. It should be noted that an interesting case, in and of itself, could be identified, especially by a patient or their family with access to social media. As such, a reasonable time gap and permission from the patient should be considered before posting.

The verification of information presented on a social media platform is largely dependent on the end user; however, institutional verification methods should exist for communications that represent and reflect on the institution. Having a green or blue ‘check mark’ next to the user profile may be helpful for patients to identify reliable sources of information, but this should be continually monitored and maintained to prevent the dissemination of misinformation.

*Physicians might consider having separate social media accounts for personal and professional purposes, and employed physicians may be subject to their institution’s social media policies. Physicians should consider social media as an extension of their identity and, therefore, appropriately conduct themselves.*

Surgeons—and all healthcare providers—are people, entitled to a personal life outside of the hospital, and for many people this includes social media activity. 60–90% of physicians have some sort of social media presence, with non-academic surgeons reporting the highest use: 71.9%, compared with 29.5% of academic surgeons [13]. However, social media and the internet should be seen as an extension of real life and not a separate entity. As such, the same expectation of professionalism that pertains to a surgeon’s conduct within *and outside* of the hospital extends to social media.

Those using social media should consult their institution for specific rules regarding their online behavior. The following include some suggestions that could support professional and ethical behavior online:

- Consider the use of separate personal and professional accounts online.
- Refrain from using professional titles for personal-only accounts.
- Consider the use of privacy features for accounts, and especially for personal accounts.
- Consider the ability of patients and colleagues to find and study a social media profile, and how this may impact patient care, job opportunities, and interpersonal relationships in the workplace.
- Consider the unique nature of the provider-patient relationship, and maintain the same separation of professional versus personal relationship with a patient online as one would in person. Consider not ‘friending’ or ‘following’ patients.
- Consider providing the primary link for data shared through social media, and encourage others to seek primary data in their quest for medical or scientific information.
- Remember that anything posted on social media remains on the Internet forever.
- Social media conduct should be subject to the same professionalism as in “real life.”
- For the physician’s privacy and safety, consider a non-identifiable name for a separate personal social media account.

## Patient use of social media

*Social media is an important tool in health information dissemination, but should not be the patient’s only form of health information.*

As of 2024, it is estimated that 62.3% of the world’s population are social media users [14], with as many as 80% of internet users searching for health-related information [15]. Articles are noting the uptrend in social media use for the dissemination of medical information, with support for continued future use as a tool beyond the pandemic [7, 8]. Increasingly, patients have been utilizing “virtual communities” both as a resource and as a support system with the potential for significant benefits, as peer influence and community support have the most impact on healthcare decisions [15]. However, the detrimental effects of disseminating inaccurate health information require further oversight and adjustments to account for all levels of medical literacy. Even younger, more educated adults, who are technologically adept, might have limited access to digital media due to socioeconomic constraints. This can result in a barrier to entry compared to a traditional in-person consultation. In addition to overall access, the privacy allotted by using social media for health information can be used for stigmatizing conditions such as sexually transmitted illnesses (STIs) and mental health issues, which would otherwise have remained under-addressed [15]^.^ Social media user groups for individual health conditions related to ICD-10 non-communicable diseases number more than 757 on Facebook when reviewed in 2009 [16]. However, there is often no official verification or credentialing needed to post educational content. In a study of 21 plastic surgery-related hashtags, only 17.8% of posts were from American Board of Plastic Surgery (ABPS) and Royal College of Physicians and Surgeons of Canada (RCPSC) board-certified plastic surgeons, with the remainder of posts composed by a variety of other surgical and medical specialties, dentists, spas, and hair salons [17].

Rather than using social media as a replacement for healthcare professionals, it more often appears complementary to the expert opinion, with patients using it to seek additional first-person experiences, as well as social comparison, and to fulfill emotional needs [18]. SM represents a safety net for patients with limited or no access to healthcare, such as those in marginalized populations, because health information may be more accessible in their native language than with the cultural barriers encountered in traditional offices. When used to find peers with similar chronic conditions to identify commonalities, patients overall felt more engaged with their healthcare provider, using in-person consultations to validate information, while feeling more empowered to participate in their care [19]. However, it is important to recognize confirmation bias as a risk for filtered content selected for patients through social media platforms [15]. Patients could utilize images and videos outlining the results to either convey a realistic sense of what is to come or, if not properly reviewed, come away with unrealistic expectations of heavily edited images [20]. Platforms such as YouTube that allow for videos for first-hand accounts were found to be more effective for communicating emotional responses and coping mechanisms, prompting more behavioral changes [21]. With the use of social media and targeted feeds, careful attention to maintaining the privacy of patients is pivotal, as it can remain a safe place to maintain “normalcy” without fear of disclosing personal information such as chronic illnesses to acquaintances who may follow them online [22].

Social media can also rapidly disseminate updates on an interactive platform in public health campaigns with evidence-based findings to combat the spread of misinformation. Careful attention must be paid to avoid losing those who lack access to social media. Social media should be used as an enhancement to traditional methods of public communication. Increasingly, it is being used both as a way of branding and marketing an institution for the new recruitment of patients who may be inaccessible by traditional methods, by providing verified health information [6]. Social media is increasingly being utilized as a method by which patients select practitioners in fields such as esthetic surgery, where the Google ranking of searches correlates with the number of social media followers rather than other factors such as training institution or years of experience [23].

In a study of discussion boards from the 15 largest Facebook groups dedicated to diabetes, with members ranging from 1,107 to 61,957, 65.7% of posts were coded as providing information and personal experiences, followed by 28.8% that provided peer support, and 26.7% were composed of advertisements. Despite that lack of fact-checkers combined with the relative anonymity of contributors, there were overall more positive patient-focused outcomes from forums dedicated to diabetes management, with rare inaccurate recommendations in only 3% of posts [24]. There is a balance where the positive effects of patient social media utilization with improved well-being and control must be recognized while minimizing the risks of privacy invasion, promotional targeting, negative effects on well-being, and social media addiction [18]. Care must be taken, as anything posted becomes a permanent part of the digital record, and further regulatory practices and education are needed for clinicians to maintain patient privacy if these modalities are increasingly incorporated in clinical care [25]. Overall, the evidence suggests that patients with active social media use endorse an increased understanding of their health, which contributes to proactive management, although there is a continued need for verified trustworthy sources to avoid misinformation and for patients to understand the overall “usefulness” of the information provided by social media [26].

*Patients’ privacy should be respected, and their social media accounts should not be used as additional information for healthcare providers caring for them.*

Healthcare practitioners should provide the same level of respect and privacy toward a patient’s online identity as they would like to receive. In general, providers should refrain from searching for patient information online. In a sense, personal information shared on social media could be considered as a form of identifiable protected health information. As per the US Department of Health and Human Services Health Insurance Portability and Accountability Act of 1996 (HIPAA), a basic and enduring principle is that circumstances in which an individual’s protected health information may be used or disclosed should be limited [27]. However, there are limited circumstances in which a patient’s online identity could be impactful to their care and well-being. For example, if an incapacitated patient arrives in the emergency room without contact information available for next of kin, an online search could provide contact information for this special emergency situation. That being said, if social media is going to be used to glean information about a patient, this must be disclosed to the patient as soon as possible and should follow institutional guidelines as applicable.

*Explicit consent from both sides must be obtained prior to any picture or video being taken for use on social media.*

As mentioned, social media is used by a huge proportion of the modern population. Patients, as people, have the right to share their information. However, patients should not share protected information of other patients, and should not share recorded media of healthcare practitioners without their consent. Unfortunately, covert recording of healthcare practitioners, especially if shared without full context, can be damaging to individual providers, hospitals, and the institution of healthcare overall. Healthcare practitioners should have the right to consent or not to the streaming, recording, or sharing of their care delivery by patients and visitors, pending individual institutional guidelines. All healthcare providers have a right to refuse audio or video recording of their patient care interactions. There must be verbal or written consent from both sides when any photography or video is done in the physician–patient setting. When patients take pictures or videos, there must be consent from both sides, even if the photos are for educational use, such as interesting case discussions. Any such images must still be de-identified to protect patient and physician privacy. There are no states that have a statute of limitations on when a video or photo can be posted; instead, these waiting periods are guided by HIPAA, state medical boards, and institutions.

## Societal impact of social media

*Use of social media for trial recruitment and crowdsourcing information should be guided by the principles of patient autonomy (privacy), non-maleficence, and justice (access).*

Crowdsourcing, a term meaning to gain the collective resources of a group, has become a way for physicians to obtain information in healthcare, with platforms like Sermo and Medscape often surveying physicians about their best practices based on case studies. While crowdsourcing is an excellent way of obtaining information, its validity remains undetermined, and the collective experience of a group may not necessarily be the right answer, even though it could be the best one, given that medicine is rarely black and white.

Another utilization of crowdsourcing is in clinical trial enrollment, where social media is used as a method of advertising. The concept of coercion arises when influencers are used to convince healthy participants to enroll when they might not have done so otherwise. However, social media provides a large amount of population data for researchers to mine. Everything from a person’s birth date to their Spotify playlist can be analyzed. The ethical principle of justice should be observed such that participants have other ways to find out about, and enroll in, clinical trials that are advertised on social media, i.e., phone, email, printed mail, for those without access to computers or who lack technological literacy.

*Social media should not be used for patient–provider communication. Only secure, trackable electronic medical record (EMR) platforms should be used.*

In future, various forms of social media will increasingly be used for interactions between patients and providers as younger providers become more comfortable with navigating the medium. As early as 2011–2012, medical students were polled, with 92% owning a Facebook account and 97% logging on multiple times a day, with the majority, 72%, reporting that they used one profile, thus combining their personal and professional lives. Of those students queried, 60% endorsed that if they were directly asked for medical advice by a patient via Facebook they would explain to the patient in person that the forum is an inappropriate means to communicate concerns, though there was some cognitive dissonance as 44% reported that they would carry this out; this suggests the need to develop further policy guidelines and incorporation of content to help providers proactively navigate the ethical quandaries presented with social media platforms [28].

On one end, healthcare organizations may implement cookies to track user behavior or even allow for logins via a patient’s social media profile, thus resulting in an organization being given further access to user data. Privacy-enhancing technologies can also be used to help protect a user’s privacy with data anonymization and communication encryption with technology that can alert users of privacy threats and enact measures to prevent the disclosure of personal information. Beyond single provider–patient interactions, user-generated data that are appropriately de-identified could also be used to identify outbreaks and hotspots for surveillance and target areas for public service campaigns. These large datasets would be a valuable resource for population health studies, although more work would be needed to interrogate the accuracy of AI-generated healthcare data derived from social media via a standardized system [29].

Thus, other than being used as marketing and educational tools, social media platforms might also be utilized for two-way communication. Careful consideration is required from both parties regarding the permanence of the digital record and awareness of privacy settings. In future, more research and further developments in AI and machine learning (ML) could assist providers in efficiently utilizing the massive amounts of user-generated data to focus on the most effective measures to protect patients, both in promoting early diagnosis and in identifying public health trends.

*Excessive use of social media may impact patient and provider mental health and wellness.*

Many note concerns that social media is detrimental to mental health, including increased depressive symptoms, loneliness, and addictive behavior toward screen time [30, 31]. Further, screen time among medical students, whether for leisure or study/work, is associated with delayed bedtime, shorter sleep duration [32] and poorer sleep quality [33]. Beyond mental health, increased screen time has been associated with poor health effects: high blood pressure, obesity, poor stress regulation, and insulin resistance [31]. Though many of these cross-sectional study populations may not be entirely generalizable to practicing physicians, healthcare providers should still take note of some of the detrimental effects of social media and the risks of continued screen exposure masquerading as important for professional work, especially when this extends beyond normal working hours.

On the other hand, other cross-sectional studies have found that self-esteem can be boosted by an online presence [34]. Further, social media can provide a sense of support and community among providers, whether through collaboration or a forum for advocacy and mentorship [3]. As with most things in life, when used in moderation, social media can enhance a provider’s career and, as such, should not be outright avoided or banned.

*Social media may have a role in advocacy and promotion of societal health.*

Though the classic non-clinical roles of a healthcare practitioner include researcher and educator, advocacy is an important responsibility for our profession as a whole. Physicians should promote health and well-being, and this can extend beyond clinical practice. This is not a new construct. Although most physicians recognize Rudolf Virchow for his eponymous thrombotic triad, he was also an epidemiologist and once said, “Medicine is a social science, and politics, nothing but medicine at a larger scale.” Physicians and healthcare practitioners as a whole hold a powerful voice in modern society, and this can, and *should*, be used to promote overall societal health. The Society of American Gastrointestinal Surgeons (SAGES) has strategically utilized various platforms, including social media, to disseminate clinical guidelines to the public for these purposes; these guidelines are downloaded worldwide for education and training purposes, adding to the promotion of population health.

The American College of Surgeons notes that surgeons should “advocate for strategies to improve individual and public health through communication with government, healthcare organizations, and industry” [11]. Social media provides a platform that can be used for this advocacy, though its use should fall within institutional guidelines and reflect that the views pertain to the individual provider, rather than their institution as a whole.

*AI may change the role of social media, and the evolution should be monitored.*

With the continued and growing use of social media, there is a potential for artificial intelligence and machine learning to sift through the vast volume of data for more effective remote patient monitoring. Chatbots are being utilized to simulate human interactions to assist with long-term patient monitoring and freeing up time for staff by managing simple tasks such as responding to a panel of frequently asked questions and scheduling appointments 24/7. User-generated data in conjunction with AI could be implemented to filter out unqualified or unverified sources and decrease the spread of misinformation, while ML could assist in tailoring content into personalized patient feeds (similar to YouTube and Facebook) to improve user engagement. Companies have also been utilizing sentiment analysis for consumer-generated feedback; application of this in the healthcare realm could analyze patient posts to highlight concerning language and alert a provider to provide more immediate attention. AI can also be used for user education and training in protecting personal information online and to serve as personalized tutors, where it is already being utilized in other fields for simulations, allowing for practice in a safe, structured environment, tracking progress, and allowing for additional courses for identified weak spots [29].

## Summary statements

1. Physicians’ use of social media for self-promotion or marketing is not inherently unethical, but should be carefully considered and conflicts of interest disclosed.
2. Physicians should include a visible disclosure that their views do not necessarily represent the institution, organization, or society, unless otherwise stated by the institution, organization, or society.
3. Social media used for educational purposes should be done with institutional oversight. Institutions and major surgical societies should dedicate staffing for policy-making, monitoring, and approval of tagged social media statements.
4. Physicians’ use of social media for education and public dissemination should be guided by HIPAA to protect patient and provider confidentiality. Posts should be subject to the same level of accuracy and evidence-based practice as would be expected of a medical professional offline.
5. Physicians should consider having separate social media accounts for personal and professional purposes, and employed physicians may be subject to their institution’s social media policies. Physicians should consider social media as an extension of their identity and, therefore, appropriately conduct themselves.
6. Social media is an important tool in health information dissemination, but should not be the patient’s only form of health information.
7. Patients’ privacy should be respected, and their social media accounts should not be used as additional information for healthcare providers caring for them.
8. Explicit consent from both sides must be obtained prior to any picture or video being taken for use on social media.
9. Use of social media for trial recruitment and crowdsourcing information should be guided by the principles of patient autonomy (privacy), non-maleficence, and justice (access).
10. Social media should not be used for patient-provider communication. Only secure, trackable EMR platforms should be used.
11. Excessive use of social media may impact patient and provider mental health and wellness.
12. Social media may have a role in advocacy and promotion of societal health.
13. AI may change the role of social media, and the evolution should be monitored.
